# The Relationship between Platelet Count and Host Gut Microbiota: A Population-Based Retrospective Cross-Sectional Study

**DOI:** 10.3390/jcm8020230

**Published:** 2019-02-10

**Authors:** Hee-Young Yoon, Han-Na Kim, Su Hwan Lee, Soo Jung Kim, Yoosoo Chang, Seungho Ryu, Hocheol Shin, Hyung-Lae Kim, Jin Hwa Lee

**Affiliations:** 1 Department of Internal Medicine, College of Medicine, Ewha Womans University, Seoul 07804, Korea; bluecat-s@hanmail.net (H.-Y.Y.); crystalkim38@gmail.com (S.J.K.); 2 Medical Research Institute, Kangbuk Samsung Hospital, Sungkyunkwan University, School of Medicine, Seoul 03181, Korea; hanna147942@gmail.com; 3 Division of Pulmonology, Department of Internal Medicine, Institute of Chest Diseases, Severance Hospital, Yonsei University College of Medicine, Seoul 03722, Korea; hihogogo@naver.com; 4 Center for Cohort Studies, Total Healthcare Center, Kangbuk Samsung Hospital, Sungkyunkwan University, School of Medicine, Seoul 04514, Korea; yoosoo.chang@samsung.com (Y.C.); sh703.yoo@samsung.com (S.R.); 5 Department of Occupational and Environmental Medicine, Kangbuk Samsung Hospital, Sungkyunkwan University, School of Medicine, Seoul 03181, Korea; 6 Department of Family Medicine, Kangbuk Samsung Hospital, Sungkyunkwan University School of Medicine, Seoul 03181, Korea; hcfm.shin@samsung.com; 7 Department of Biochemistry, College of Medicine, Ewha Womans University, Seoul 07804, Korea

**Keywords:** gut microbiota, 16S RNA, platelet, thrombocytosis, *Faecalibacterium*

## Abstract

Platelet count reflects the severity and prognosis of multiple diseases. Additionally, alterations in gut microbiota have been linked to several chronic diseases. The purpose of this study was to investigate the association between gut microbiota and platelet count. We selected 1268 subjects with fecal 16S RNA gene sequencing data from a Healthcare Screening Center cohort. Based on the third quartile of platelets (277 × 10^9^/L), we compared the gut microbiota between the upper quartile (*n* = 321) and lower three quartiles groups (*n* = 947). The upper quartile group had lower alpha diversity based on observed amplicon sequence variants (*q* = 0.004) and phylogenetic index (*q* < 0.001) than the lower three quartiles group. Significant differences were also found in the weighted UniFrac distance (*q* = 0.001) and Jaccard dissimilarity (*q* = 0.047) beta diversity measures between the two groups. Compared with the lower three quartiles group, the upper quartile group exhibited decreased relative abundances of the genus *Faecalibacterium*, which was also inversely correlated with the platelet count. Increased platelet count was associated with reduced diversity in gut microbiota and lower abundances of *Faecalibacterium* with beneficial gut bacteria spices *F. prausnitzii*, suggesting that an increased platelet count, even within normal range, may adversely affect gut microbial diversity and composition.

## 1. Introduction

Humans consistently interact with their microbiota, which is defined as the collection of microorganisms living inside and on the human body. The bacteria of the microbiota and their genomes are collectively referred to as the microbiome [1,2]. The human gut contains the greatest number and density of bacteria in the human body, and its microbiota is host-specific based on heritable components [3] and is modified by acquired factors such as diet, drugs, surgery, and aging [4,5,6,7]. The gut microbiota plays important roles in the development and progression of numerous medical conditions, including obesity [8], inflammatory bowel disease (IBD) [9], cardiovascular disease [10,11], and neurodegenerative diseases [12]. The recent terms “gut‒brain axis” and “gut‒lung axis” indicate that gut microbiota can influence multiple organs and systems by producing metabolites and chemicals, and causing the release of inflammatory cytokines [13,14].

Thrombocytosis is mainly derived from secondary causes with the exception of essential thrombocytosis, which is due to clonal thrombopoiesis. Not only acute conditions such as infection, inflammation, and blood loss, but also chronic conditions (e.g., iron deficiency, chronic inflammatory or infectious diseases, malignancy) contribute to reactive thrombocytosis [15]. In addition, platelets regulate the immune system by releasing pro-inflammatory factors and activating innate immunity [16]. Although thrombocytosis has been demonstrated to predict the prognosis of several pathologic conditions [17,18,19,20], the impact of relative increases in platelet count within the normal limit on the human body remains unclear. Because the gut microbiota is a sensitive marker of several diseases, reflecting an individual’s immune system and inflammatory status, we hypothesized that small changes in platelet count might lead to changes in the gut microbiome.

We previously demonstrated that the neutrophil-to-lymphocyte ratio and inflammatory markers were associated with the gut microbiome in a Korean population [21]. Here, we aimed to evaluate the association between the gut microbiota and platelet count in a Korean population using health screening data.

## 2. Materials and Method

### 2.1. Study Population

**Figure 1:**
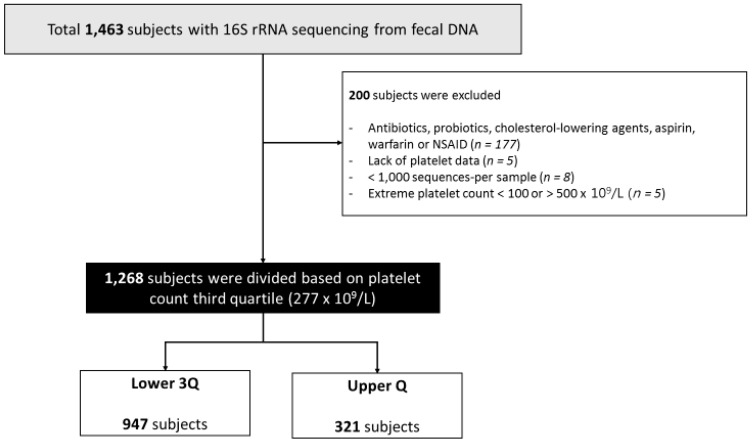
Flowchart of subjects’ enrollment. NSAID, non-steroidal anti-inflammatory drugs; Lower 3Q, <75th percentile for platelet count; Upper Q, ≥75th percentile for platelet count.

We screened 1463 Korean men and women between 25 and 78 years old who visited Kangbuk Samsung Hospital Healthcare Screening Center in the Republic of Korea for a comprehensive annual or biennial physical examination from June to September 2014 (Figure 1).

Among 1463 subjects with fecal DNA samples for 16S rRNA sequencing, 195 subjects were excluded for the following reasons: medications that could affect the gut microbiome and/or platelet function (*n* = 182), lack of baseline platelet count (*n* = 5), or less than 1,000 sequences per sample (*n* = 8). Additionally, five subjects were excluded because their platelets were not within the newly devised reference range (between 100 and 500 × 10^9^/L) based on the distribution of platelet counts excluding outliers. Finally, 1268 subjects were included in our study.

### 2.2. Study Design

We assessed correlations between platelet count and the relative abundances of gut microbiota. Our subjects were divided into two groups, the lower three quartiles (3Q; *n* = 947) and the upper quartile (*n* = 321) based on third quartile platelet count (3Q = 277 × 10^9^/L). The diversity and composition of the gut microbiota were compared between the upper Q and lower 3Q groups. Because the normal range of platelet count varies by sex [22,23], subgroup analysis was performed using the third quartile platelet count according to sex (268 × 10^9^/L for men and 294 × 10^9^/L for women).

Demographic data, laboratory findings, and medical and diet histories were retrospectively reviewed through medical records and questionnaires taken at the time of sample collection. This study was approved by the Institutional Review Board of Kangbuk Samsung Hospital (KBSMC 2013-01-245-12) and written informed consent was provided by all subjects.

### 2.3. DNA Extraction and 16S Gene rRNA Sequencing for Bacterial Communities

Fresh fecal samples were obtained immediately after defecation and stored at −70 °C within 24 h until DNA extraction. Genomic DNA was extracted using the MO Bio PowerSoil^®^ DNA Isolation Kit (MO BIO Laboratories, Carlsbad, CA, USA) in accordance with the manufacturer’s instructions. The methods used to amplify and sequence the DNA were detailed in our previous study [24]. Briefly, the universal primers 341F and 805R were used to amplify genomic DNA in the variable V3 and V4 regions of the 16S rRNA genes. The 16S rRNA sequencing of all samples was performed with the read length of 300 bp paired-end using the Illumina MiSeq platform (Illumina, San Diego, CA, USA) with the manufacturer’s specifications [25].

### 2.4. Compositional Analysis of 16S rRNA Gene

The sequence quality control and feature table construction were performed using DADA2 [26] of QIIME2 plugins (version 2018.08, https://qiime2.org) [27,28]. Low quality sequences and chimeric sequences were excluded. The amplicon sequence variants (ASVs) were produced by denoising with DADA2 and regarded as 100% operational taxonomic units (OTUs) [29]. After denoising, the paired-sequences were merged and, after that, the chimeras were removed. We created the feature table, including the abundance table and the representational sequence file. The taxonomic classification was performed using a pre-trained Naïve Bayes classifier and the q2-feature-classifier plugin. This classifier was trained on the V3–V4 region containing the gene from 99% OTUs files in the Greengenes 13_8 release of 16S rRNA gene sequences [30].

### 2.5. Statistical Analysis

All continuous variables were presented as mean ± standard deviation (SD) and categorical variables were expressed as number (%). QIIME2 (version 2018.08) was used to analyze the diversity and composition of gut microbiota between groups [29]. Before diversity analyses, the feature tables were rarefied with 1000 sample depth evenly by random subsampling. Alpha diversity indices, which measures the number of distinct ASVs in each sample, was expressed using the actual number of different taxa observed in a sample as the non-phylogenetic index (“Observed ASVs”) and a phylogenetic diversity (PD) measurement, Faith’s PD, which incorporated phylogenetic difference between ASVs [31]. Alpha diversity was also measured using the following two non-phylogenetic methods: the Shannon index, which is measured by accounting for both evenness and richness [32], and the Pielou’s evenness, which quantifies how equal the community is numerically [33]. The difference in alpha diversity between groups was calculated using the Kruskal‒Wallis test. The beta diversity between groups was assessed using with following three methods: unweighted UniFrac distance as phylogenetic and qualitative index; weighted UniFrac distance as phylogenetic and quantitative index for abundance differences [34]; Jaccard dissimilarity as non-phylogenetic and qualitive index for presence/absence differences [35]. Differences in beta diversity between platelet groups was compared using pairwise permutational multivariate analysis of variance (PERMANOVA with 999 permutations) [36]. For composition analysis, taxa with low abundance (less than 10%) were filtered. Analysis of composition of microbiome (ANCOM) in QIIME2 was used to compare the log-ratio different abundances of gut microbial taxa in the upper and lower 3Q groups [37]. The correlation between the abundance of taxa and platelet count and comparison of the abundance of taxa between the upper and lower 3Q groups were assessed using multivariate association with linear models (MaAsLin, version 1.0.1, http://huttenhower.sph.harvard.edu/maaslin) software package [38] for R (version 3.5.1, URL http://www.R-project.org). Analyses included covariate adjustments for age, sex, smoking status, and body mass index (BMI), all of which could affect gut microbiome composition [39,40,41,42]. All analyses using MaAsLin were performed with the default settings and presented as covariate-adjusted coefficients (CE). The differentially abundant taxa between the two groups at different taxonomy levels were presented as W-statistics.

Additionally, microbial community function was evaluated by predictive metagenome (microbial DNA) analysis using PICRUSt (Phylogenetic Investigation of Communities by Reconstruction of Unobserved States) [43]. PICRUSt is a developed phylogeny-based computational tool that predicts the functional capacity of microbial communities by correlating the species present to reference databases of microbial genomes. We performed PICRUSt with de-novo variants according to a recent manual (https://github.com/LangilleLab/microbiome_helper/wiki/PICRUSt-Tutorial-with-de-novo-Variants). DADA2 variants were normalized using the 16S rRNA copy number, and KEGG (Kyoto Encyclopedia of Genes and Genomes) orthologs (KOs) were predicted. Results that aggregated to level three of the KEGG analysis module were further explored with STAMP (statistical analysis of taxonomic and functional profiles) version 2.1.3 [44], using two-group analysis module. The resulting *p*-values were corrected for multiple comparisons on the number of pathways using FDR (Benjamini‒Hochberg, *q*-value).

## 3. Results

### 3.1. Baseline Characteristics

Of the total 1268 subjects (mean age: 45.4 years, men: 62.1%), 947 and 321 subjects were classified into the lower 3Q and upper Q groups, respectively (Table 1).

The upper Q group had fewer men than the lower 3Q group. Additionally, the upper Q group had more total white blood cells (WBCs), neutrophils, and basophils, but lower levels of monocytes, hematocrit, serum iron, and ferritin compared to the lower 3Q group. There were no significant differences in comorbidities or nutritional status between the two groups (Supplementary Tables S1 and S2).

Because the sex distribution between the two groups was different, we performed sub-group analyses according to sex using sex-specific third quartiles of platelet count (268 × 10^9^/L for men and 294 × 10^9^/L for women). In men, WBC and basophil counts were significantly different between the lower 3Q (*n* = 589) and upper Q (*n* = 198) groups (Supplementary Table S3). In women, the upper Q group (*n* = 121) had a higher BMI, higher levels of WBC and basophils, and lower levels of hematocrit, serum iron, and ferritin compared with the lower 3Q group (*n* = 360).

### 3.2. Comparison of Alpha Diversity between the Upper Quartile and Lower Three Quartiles Groups

**Figure 2:**
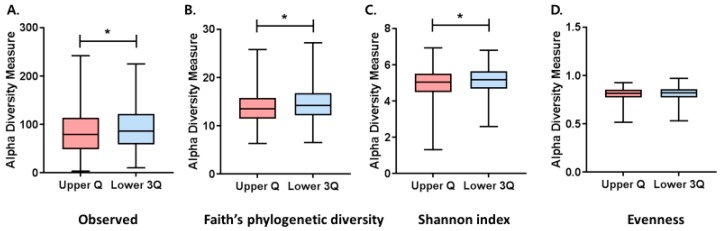
Comparison of alpha diversity indexes between the upper and lower three quartiles groups. (**A**) Observed amplicon sequence variants (ASVs), (**B**) phylogenetic diversity, (**C**) Pielou evenness, and (**D**) Shannon’s index. Lower 3Q, <75th percentile for platelet count; Upper Q, ≥75th percentile for platelet count. * *q* < 0.05.

The upper Q group had significantly lower alpha diversity measurements including observed ASVs (*q* = 0.004), PD (*q* < 0.001) and Shannon index (*q* = 0.002) compared to the lower 3Q group (Figure 2). However, the evenness was not significantly different between the two groups (*q* = 0.254).

Subgroup analyses of men showed that alpha diversity in the upper Q group as measured by observed ASVs (*q* = 0.048), PD (*q* = 0.007), and the Shannon index (*q* = 0.009) was also lower than in the lower 3Q groups (Supplementary Figure S1). Additionally, the evenness between the two groups approached significance (*q* = 0.078). In contrast, in women, no significant differences in alpha diversity between the two groups were observed (Supplementary Figure S2).

### 3.3. Comparison of Beta Diversity between the Upper Quartile and Lower Three Quartiles Groups

Significant differences in beta diversity between the upper Q and lower 3Q groups were identified as measured by unweighted UniFrac distance (*q* = 0.001) and Jaccard dissimilarity (*q* = 0.017), but not by weighted UniFrac analysis (*q* = 0.156; Table 2 and Supplementary Figure S3).

Subgroup analyses of men showed that beta diversity measured by unweighted (*q* = 0.003) and weighted UniFrac distance (*q* = 0.020) was also significantly different between the two groups (Supplementary Figure S4), and the difference in beta diversity based on Jaccard dissimilarity approached statistical significance (*q* = 0.076). In contrast, the beta diversity measurements between the upper Q and lower 3Q groups were similar in women, with only a trend toward significance for the unweighted UniFrac distance (*q* = 0.052; Supplementary Figure S5).

### 3.4. Comparison of the Microbial Composition between the Upper Quartile and Lower Three Quartile Groups

Significant differences in relative abundances between the upper Q and lower 3Q groups were assessed from phylum to species level using the ANCOM method (Table 3). The upper Q group had significantly reduced abundance of Clostridia class, Clostridiales order, Ruminococcaceae family, and *Faecalibacterium* genus compared to the lower 3Q group. The W is interpreted as follows: the “W = 10” of Clostridia indicates that the class was detected to be significantly different relative to 10 other classes between the two groups.

Among men, no significant differences in the relative abundances of taxa between the upper Q and lower 3Q were identified. However, the upper Q group in women showed a remarkably decreased abundance of the Ruminococcaceae family and *Faecalibacterium* genus compared to the lower 3Q group (Supplementary Table S4). In contrast, the Chloroplas class and Aeromonadales order was increased in the female upper Q group, while the Mollicutes class was decreased in the female upper Q group compared with the female lower 3Q group.

### 3.5. Correlation between Platelet Count and Gut Microbiota

Based on a MaAsLine analyses adjusted for age, sex, smoking status, BMI, total WBC count, and hematocrit in all subjects, the relative abundance of several bacteria decreased as the platelet count increased (Table 4). The platelet count negatively correlated with the genera *Faecalibacterium* (CE: −0.00022, *q* = 0.0016).

### 3.6. Comparison of the Functional Microbial Composition between the Upper Quartile and Lower Three Quartiles Groups 

We compared functional profiles generated by PICRUSt between the upper Q and the lower 3Q groups. The predicted functions of the gut microbiota in the upper Q and lower 3Q groups were similar based on KEGG ortholog composition, with only subtle statistical differences observed based on unadjusted *p*-values. Based on level two profile analysis, PICRUSt predicted a higher abundance of genes in the ‘Infectious diseases’ KEGG pathway in the upper Q group compared to the lower 3Q group. Based on level three profile analysis, genes related to the ‘Nitrogen metabolism’ pathway were highly enriched in the upper Q group, while the pathway related to “Glycosphingolipid biosynthesis-lacto and neolacto series” was enriched in the lower 3Q group.

Subgroup analysis of men only indicated that in the lower 3Q group, there were very subtle increases in the genes involved in the ‘Glycosphingolipid biosynthesis–lacto and neolacto series’ pathway that were significant following adjustment compared with the upper group (difference: 4.50 × 10^−7^, 95% confidential interval: 2.22 × 10^−7^ to 6.78 × 10^−7^, adjusted *p* = 0.038; Supplementary Figure S6). Among women, we found that several pathways related to the immune system, infectious diseases, and energy metabolism were higher in the upper Q group than in the lower 3Q groups; however, these differences were only supported by unadjusted *p*-values.

## 4. Discussion

This large cohort study demonstrated that increased platelet count was associated with decreased diversity of the gut microbiota in a Korean population. The gut microbiota composition also changed according to platelet level. In particular, there was a negative correlation between platelet count and genus *Faecalibacterium*. In subgroup analyses according to sex, diversity was significantly different between the upper Q and lower 3Q groups in men, whereas only differences in composition were observed between the two groups in women. Comparative analysis of functional composition revealed some differences between the two groups, but these differences were not statistically significant.

In this study, platelet count was inversely correlated with the relative abundance of *Faecalibacterium prausnitzii*; the sole known species of *Faecalibacterium* is one of the major butyrate producers of gut microbiota [45]. Among short-chain fatty acids (SCFAs), microbiota-induced fermentation products such as butyrate are a main energy source for colonocytes and can promote a healthy gut through anti-inflammatory action [46]. *F. prausnitzii* plays a beneficial role in the intestine by secreting butyrate with unidentified metabolites [47]. Furthermore, because its abundance is reduced in several intestinal diseases, including IBD and colon cancer [47,48], *F. prausnitzii* has potential as a biomarker for gut health. It is possible that the association between *F. prausnitzii* and platelets may be the reason that platelet count is associated with IBD prognosis [49]. Interestingly, elevated platelet counts are related to prognosis in diabetic patients [50,51], in whom the abundance of *F. prausnitzii* is lower compared with non-diabetic patients and inversely correlated with inflammatory markers such as interleukin-6 (IL-6) and hs-CRP [52]. Given the association between platelet count and several diseases in which the abundance of *F. prausnitzii* correlates with prognosis or disease severity, platelet count might be suppressed by the anti-inflammatory activity of *F. prausnitzii* [46]. Consistent with this, a decrease in IL-6, which promotes platelet production [53], was observed after treatment with *F. prausnitzii* in a mouse model [54].

Our study demonstrated that the alpha diversity in the upper Q group was reduced compared with the lower 3Q group. This finding suggests that the composition of the gut microbiota might be affected by platelet count and/or could contribute to thrombocytosis. Reduced diversity of the gut microbiota often leads to outgrowth of a few species and decreased resilience, which can be unhealthy [55,56]. Several chronic diseases including obesity, IBD, diabetes, and atopic eczema, with increased prevalence in recent decades have been linked to low diversity of the gut microbiota [1,57,58,59]. In addition, low gut microbiome diversity is associated with increased mortality in some clinical conditions such as allogenic hematopoietic stem cell transplantation and graft-versus-host disease [60,61]. Taken together, increased platelet count could represent a reduction in gut diversity.

According to our results, expression of genes related to the ‘Glycospingolipid biosynidesis–lacto and neolacto series’ pathway was increased in the male lower 3Q group compared with the male upper Q group. Glycospingolipids (GSLs) are a subtype of glycolipids found in the cell membrane of human and bacterial cells that comprise a ceramide backbone covalently bonded to a glycan moiety. GSL biosynthesis is a stepwise process, characterized by first adding sugars to ceramides followed by glycan growth [62]. Among GSLs during this synthesis process, GM1 [63], GD1a, GD1b, and GT1b [64] suppress platelet-derived growth factor (PDGF)-dependent cell growth and receptor tyrosine phosphorylation. PDGF, which is released from platelets, stimulates the proliferation of megakaryocytes in vitro [65], while PDGF knockout leads to thrombocytopenia in mouse embryos [66]. Furthermore, there are several clinical conditions related to thrombocytosis as well as essential thrombocythemia that lead to increased PDGF levels [67,68,69,70]. Overall, gut microbiota might induce thrombocytosis by generating GSLs that inhibit PDGF activity.

Although a normal platelet count generally ranges from 150 to 450 × 10^9^/L [71], there are sex-specific differences in normal platelet count. Specifically, women have a slightly higher platelet count than men [23]. In our study, the greater number of women in the upper Q group was attributed to the higher third quartile value in women than in men (294 [women] vs. 268 × 10^9^/L [men]). This imbalanced gender distribution also resulted in differences in hematocrit, iron, and ferritin between the upper Q and lower Q groups because the women in our study were mostly young, healthy, premenopausal women with potential iron deficiency anemia (IDA). In IDA women, platelet counts are inversely correlated with hematocrit and iron [72,73], which supports our findings. However, these factors are unlikely to have significantly impacted our results because the microbial diversity differences between the upper and lower groups were only found in men and all subjects and not in women. The lack of statistical differences in women could be attributed to the relatively small number of subjects. Compositional differences between the two groups were noticeable in women, possibly due to the wider distribution of platelets (mean ± SD: 261.2 ± 56.2 (women) vs. 238.8 ± 47.6 × 10^9^/L (men), *p* < 0.001). Therefore, in women, the extreme distributional differences in platelet counts may have contributed to the significant differences observed in the composition of the microbiota.

Our study had several limitations. First, this was a single center, cross-sectional retrospective study without longitudinal follow-up data. Therefore, we could not determine whether platelet changes or gut microbiome changes occurred first. However, we assumed that gut microbiome affects platelet count through microbial functional pathway analysis. Second, our study was conducted on a relatively healthy population and therefore did not include patients with thrombocytosis meeting the common definition (>500 × 10^9^/L). We only demonstrated that a relative elevation of platelets within normal range was linked to a potentially characteristic gut environment. To investigate the association between the gut microbiome and thrombocytosis, further studies are required. Third, we identified the quantitative association between platelet count and the gut microbiome but did not confirm any qualitative association. Since platelets are functionally independent from their count [74], subjects with platelet function defects might have influenced the results of our study. To reduce this possibility, subjects receiving medications that would affect platelet function were excluded from our study. Lastly, the baseline characteristics between the upper Q and lower 3Q groups were not identical; in particular, gender distribution was varied. Those differences might have affected gut microbiome analyses. Therefore, we analyzed the diversity and composition of the gut microbiome separately in men and women. Linear correlation was also adjusted by sex, age, BMI, smoking status, WBC count, and hematocrit to control for any additional confounders.

## 5. Conclusions

In summary, the diversity and composition of the gut microbiome in patients with increased platelet counts were distinct from those with lower platelet counts. The genus *Faecalibacterium*, which contains the species *F. prausnitzii* with beneficial effects on gut, was negatively correlated with platelet count, potentially due to its anti-inflammatory function. Overall, we firstly report an association between gut microbiota and platelet count, which suggests that gut microbiota might influence platelet count systemically. Thus, platelet count could be a biomarker for detecting changes in gut microbiota, even in populations with platelet counts within relatively normal range. Additional studies are needed to confirm the antecedent association and mechanism between platelets and gut microbiota

## Figures and Tables

**Table 1:** Baseline demographics and laboratory findings between the upper and lower 3 quartiles groups.

Variables	Lower 3Q	Upper Q	Total	*p*-Value
No.	947	321	1268	
Age, years	45.7 ± 9.0	44.7 ± 8.4	45.4 ± 8.8	0.093
Male sex	634 (66.9%)	153 (47.7%)	787 (62.1%)	<0.001
Body mass index, kg/m^2^	23.6 ± 3.1	23.6 ± 3.1	23.6 ± 3.1	0.748
Smoking status				0.056
Never	505 (57.0%)	195 (64.8%)	700 (59.0%)	
Former	216 (24.4%)	58 (19.3%)	274 (23.1%)	
Current	165 (18.6%)	48 (15.9%)	213 (17.9%)	
Smoking amount, pack-years	14.4 ± 10.8	15.4 ± 13.9	14.6 ± 11.6	0.483
**Laboratory finding**				
Platelet, 10^9^/L	224.4 ± 33.5	314.9 ± 36.4	247.3 ± 52.2	<0.001
White blood cell, 10^3^/mm^3^	5.6 ± 1.4	6.4 ± 1.6	5.8 ± 1.5	<0.001
Neutrophil, %	54.9 ± 8.0	56.0 ± 7.7	55.2 ± 8.0	0.037
Lymphocyte, %	33.5 ± 7.4	34.9 ± 7.1	35.4 ± 7.3	0.167
Eosinophil, %	2.6 ± 2.1	2.4 ± 1.9	2.6 ± 2.4	0.189
Basophil, %	0.4 ± 0.3	0.5 ± 0.3	0.5 ± 0.3	0.003
Monocyte, %	6.5 ± 1.6	6.2 ± 1.5	6.4 ± 1.6	0.003
Neutrophil/lymphocyte ratio	1.7 ± 0.7	1.7 ± 0.6	1.7 ± 0.7	0.238
Hematocrit, %	42.3 ± 3.6	40.9 ± 4.0	42.0 ± 3.7	<0.001
Iron, µg/dL	122.0 ± 41.4	115.5 ± 4.5.6	120.4 ± 42.6	0.045
Ferritin, ng/mL	161.1 ± 135.1	138.0 ± 128.3	155.3 ± 133.7	0.008
C-reactive protein, mg/dL	0.1 ± 0.2	0.1 ± 0.1	0.1 ± 0.2	0.808

Data are presented as mean (standard deviation) or number (%). Lower 3Q, <75th percentile for platelet count; Upper Q, ≥75th percentile for platelet count.

**Table 2:** The statistical significances of beta diversity distances from lower three quartiles groups based on different measurement methods.

Beta Diversity Indices	Total	Male	Female
Unweighted UniFrac distance	3.472 *	2.964 *	1.598
Weighted UniFrac distance	1.696	3.074 *	1.643
Jaccard dissimilarity	1.299 *	1.167	1.059

The values are presented with the pseudo-F statistic from 999 permutation. * *q* < 0.05.

**Table 3:** The comparison of microbiome composition between the upper and lower three quartiles groups.

Level	Taxonomic Assignment	W ^a^	Normalized W ^b^
Class	k__Bacteria; p__Firmicutes; c__Clostridia *	10	0.32
Order	k__Bacteria; p__Firmicutes; c__Clostridia; o__Clostridiales *	21	0.44
Family	k__Bacteria; p__Firmicutes; c__Clostridia; o__Clostridiales; f__Ruminococcaceae *	34	0.40
Genus	k__Bacteria; p__Firmicutes; c__Clostridia; o__Clostridiales; f__Ruminococcaceae; g__Faecalibacterium *	140	0.65

* Decreased in the upper group. k, kingdom; p, phylum; c, class; o, order; f, family; g, genus; ^a^ if W = X for taxon k, then H_0k_ is rejected X times. The W statistic for a significant difference in taxa relative to other taxa at each taxa level is represented; ^b^ W statistics are normalized with each total taxa number (W statistic/total taxa number (class: 31, order: 48, family: 85, genus: 214)).

**Table 4:** The correlation between identified taxa and platelet count on MaAsLin analysis.

Order	Family	Genus	*n* Not to Zero (%)	CE	*p*-Value *	*q*-Value *
*Clostridiales*	*Ruminococcaceae*		1257	−0.00031	0.00043	0.0064
*Clostridiales*	*Ruminococcaceae*	*Faecalibacterium*	1220	−0.00022	0.00097	0.0016

CE, coefficient. * Adjusted for age, sex, body mass index, smoking status, total white blood cell count, and hematocrit. The regression CE represents the rate of change in abundance of taxa per 10^9^/L platelets.

## Supplementary Materials

The following are available online at https://www.mdpi.com/2077-0383/8/2/230/s1, Table S1: Comparison of commodities between the upper and lower three quartile groups, Table S2: Comparison of nutritional status between the upper and lower three quartile groups, Table S3: Comparison of baseline demographics and laboratory findings between the upper and lower three quartile groups within males and females, Table S4: The comparison of microbiome composition between the upper and lower three quartile groups in females, Figure S1: Comparison of alpha diversity indexes between the upper and lower three quartile groups in the males, Figure S2: Comparison of alpha diversity indexes between the upper and lower three quartile groups in the females, Figure S3: Prediction of metagenome functional content correlated with platelet count using PICRUSt, Figure S4: Beta diversity distances from the lower three quartile groups using different measurement methods in the whole subjects population, Figure S5: Beta diversity distances from the lower three quartile groups using different measurement methods in males, Figure S6: Beta diversity distances from the lower three quartile groups using different measurement methods in females.
