# Supplementary material for: The Relationship between Platelet Count and Host Gut Microbiota: A Population-Based Retrospective Cross-Sectional Study
Source: J Clin Med. 2019 Feb 10;8(2):230. doi: 10.3390/jcm8020230 (PMC6406547; doi:10.3390/jcm8020230)
Supplement: Supplementary file 1 [file jcm-08-00230-s001.pdf]

Table S1. Comparison of commodities between the upper and lower three quartile groups

Variables	Lower 3Q	Upper Q	Total	P-value
No.	947	321	1268	
Dyslipidemia	146 (15.4%)	45 (14.0%)	191 (15.1%)	0.602
Liver disease*	132 (14.0%)	33 (10.3%)	165 (13.0%)	0.111
Colon polyp	127 (13.4%)	39 (12.1%)	166 (13.1%)	0.625
Thyroid disease†	116 (12.3%)	52 (16.2%)	168 (13.3%)	0.089
Hypertension	102 (10.8%)	30 (9.3%)	132 (10.4%)	0.534
GB disease	44 (4.7%)	17 (5.3%)	61 (4.8%)	0.752
History of tuberculosis	38 (4.0%)	8 (2.5%)	46 (3.6%)	0.276
Diabetes	38 (4.0%)	7 (2.2%)	45 (3.6%)	0.173
Osteoarthritis	26 (2.7%)	11 (3.4%)	37 (2.9%)	0.666
History of malignancy‡	28 (3.0%)	13 (4.0%)	41 (3.2%)	0.441
Osteoporosis	10 (1.1%)	7 (2.2%)	17 (1.3%)	0.218
COPD	9 (1.0%)	2 (0.6%)	11 (0.9%)	0.842
Stroke	9 (1.0%)	0 (0.0%)	9 (0.7%)	0.171
Heart disease	4 (0.4%)	2 (0.6%)	6 (0.5%)	1.000
Coronary disease	4 (0.4%)	2 (0.6%)	6 (0.5%)	1.000

Data are presented as number (%).

Lower 3Q, < 75th percentile for platelet count; Upper Q, ≥ 75th percentile for platelet count

; COPD, chronic obstructive pulmonary disease

*Liver disease included hepatitis B, hepatitis C, liver cirrhosis, and fatty liver

†Thyroid disease included

‡Malignancy included breast, thyroid, stomach, liver, lung, colon, cervix, and prostate cancer

Table S2. Comparison of nutritional status between the upper and lower three quartile groups

Variables	Lower 3Q	Upper Q	Total	P-value
No.	947	321	1268	
Total energy, kcal/day	1411.5 ± 644.8	1389.3 ± 638.0	1406.0 ± 642.9	0.648
Total protein, g/day	48.5 ± 24.8	48.2 ± 25.2	48.4 ± 24.9	0.875
Total fat, g/day	28.4 ± 19.5	27.5 ± 17.9	28.2 ± 19.1	0.498
Total carbohydrate, g/day	236.8 ± 109.5	234.2 ± 111.1	236.1 ± 109.9	0.758
Total calcium mg/day	311.7 ± 210.6	306.0 ± 204.7	310.3 ± 209.1	0.720
Total phosphorus, mg/day	711.4 ± 345.9	701.3 ± 343.1	708.9 ± 345.1	0.696
Total vitamin	325.5 ± 238.7	367.8 ± 293.7	336.0 ± 253.9	0.047
Total sodium, mg/day	1621.2 ± 1043.6	1631.9 ± 1051.1	1623.9 ± 1044.9	0.892
Vitamin B1, mg/day	0.8 ± 0.5	0.8 ± 0.4	0.8 ± 0.5	0.724
Vitamin C, mg/day	68.5 ± 54.3	77.6 ± 68.4	70.7 ± 58.2	0.064
Folate, mg/day	148.0 ± 96.4	157.9 ± 109.3	150.5 ± 99.8	0.215
Retinol, ug/day	70.8 ± 61.5	71.2 ± 63.1	70.9 ± 61.8	0.939
Fiber, g/day	3.7 ± 2.2	3.8 ± 2.4	3.7 ± 2.2	0.507
Cholesterol, mg/day	167.9 ± 139.4	175.0 ± 130.9	169.7 ± 137.3	0.494

Data are presented as mean (standard deviation) or number (%).

Lower 3Q, < 75th percentile for platelet count; Upper Q, ≥ 75th percentile for platelet count

Table S3. Comparison of baseline demographics and laboratory findings between the upper and lower three quartile groups within males and females

Variables	Male			Female		
	Lower 3Q	Upper Q	P-value	Lower 3Q	Upper Q	P-value
No.	589	198		360	121	
Age, years	46.2 ± 9.2	45.1 ± 8.1	0.101	44.8 ± 8.6	44.3 ± 8.6	0.564
Body mass index, kg/m^2	24.5 ± 2.9	24.8 ± 2.9	0.173	21.7 ± 2.7	22.7 ± 3.0	<0.001
Smoking status			0.254			0.493
Never	223 (39.5%)	65 (33.3%)		310 (96.9%)	102 (94.4%)	
Former	194 (34.4%)	70 (35.9%)		6 (1.9%)	4 (3.7%)	
Current	147 (26.1%)	60 (30.8%)		4 (1.2%)	2 (1.9%)	
Smoking amount, pack-years	14.5 ± 10.9	16.1 ± 13.1	0.238	3.6 ± 3.7	5.0 ± 2.8	0.442
Laboratory finding						
Platelet, $10^9/L$	218.0 ± 31.9	300.9 ± 29.2	<0.001	236.8 ± 35.6	333.6 ± 42.3	<0.001
White blood cell, $10^3/mm^3$	5.9 ± 1.4	6.6 ± 1.6	<0.001	5.2 ± 1.3	6.0 ± 1.3	<0.001
Neutrophil, %	54.8 ± 8.1	54.2 ± 7.7	0.398	55.8 ± 8.0	56.9 ± 7.4	0.150
Lymphocyte, %	35.3 ± 7.3	35.5 ± 7.1	0.673	35.7 ± 7.5	34.6 ± 7.0	0.148
Eosinophil, %	2.8 ± 2.3	3.1 ± 2.1	0.175	2.1 ± 1.7	2.0 ± 1.2	0.448

Basophil, %	0.4 ± 0.3	0.5 ± 0.3	0.026	0.4 ± 0.3	0.5 ± 0.3	0.019
Monocyte, %	6.7 ± 1.6	6.7 ± 1.5	0.972	6.0 ± 1.5	6.0 ± 1.5	0.858
Hematocrit, %	44.1 ± 2.5	43.9 ± 2.4	0.427	38.8 ± 2.5	37.8 ± 3.4	0.003
Iron, ug/dL	126.9 ± 38.9	130.1 ± 37.6	0.392	112.5 ± 44.4	95.7 ± 49.2	0.004
Ferritin, ng/ml	212.0 ± 139.4	219.2 ± 12.16	0.492	61.6 ± 50.1	50.5 ± 42.2	0.019
C-reactive protein, mg/dL	0.1 ± 0.2	0.1 ± 0.01	0.702	0.1 ± 0.2	0.1 ± 0.1	0.482

Data are presented as mean (standard deviation) or number (%).

Lower 3Q, < 75th percentile for platelet count; Upper Q, ≥ 75th percentile for platelet count

Table S4. The comparison of microbiome composition between the upper and lower three quartile groups in females

Level	Taxonomic Assignment	W[a]	Normalized W[b]
**Class**	k__Bacteria; p__Cyanobacteria; c__Chloroplast†	2	0.07
	k__Bacteria; p__Tenericutes; c__Mollicutes*	7	0.27
**Order**	k__Bacteria; p__Proteobacteria; c__Gammaproteobacteria; o__Aeromonadales†	4	0.10
**Family**	k__Bacteria; p__Firmicutes; c__Clostridia; o__Clostridiales; f__Ruminococcaceae*	25	0.33
**Genus**	k__Bacteria; p__Firmicutes; c__Clostridia; o__Clostridiales; f__Ruminococcaceae; g__*Faecalibacterium**	51	0.27

* decreased in the upper group.

†increased in the upper group.

k, kingdom; p, phylum; c, class; o, order; f, family; g, genus; s, species

[a]If W=X for taxon k, then H_{0k} is rejected X times. The W statistic for a significant difference in taxa relative to other taxa at each taxa level is represented

[b] W statistics are normalized with each total taxa number (W statistics / total number [class: 26, order: 40, family: 76, genus: 187)

Figure S1. Comparison of alpha diversity indexes between the upper and lower three quartile groups in the males

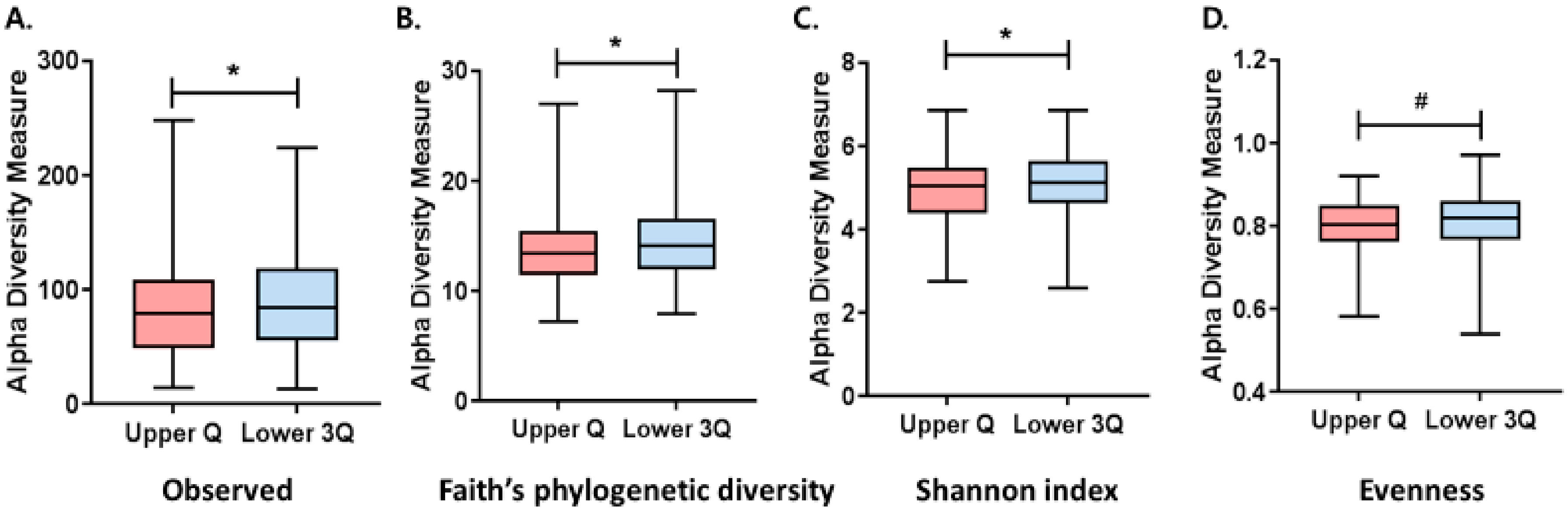

(A) Observed ASVs, (B) Faith's phylogenetic diversity, (C) Pielou's evenness, and (D) Shannon's index

ASVs, amplicon sequence variants; Lower 3Q, < 75th percentile for platelet count; Upper Q, ≥ 75th percentile for platelet count

*q<0.05, $^{\#}$q<0.1

Figure S2. Comparison of alpha diversity indexes between the upper and lower three quartile groups in the females

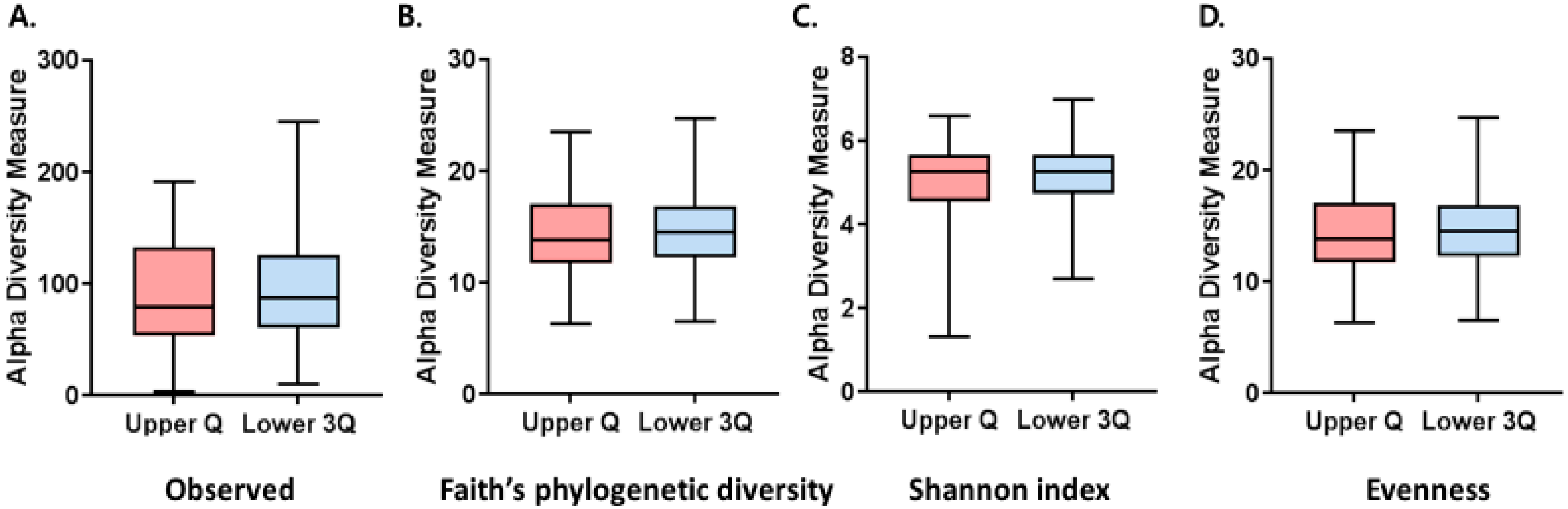

(A) Observed ASVs, (B) Faith's phylogenetic diversity, (C) Pielou's evenness, and (D) Shannon's index

ASVs, amplicon sequence variants; Lower 3Q, < 75th percentile for platelet count; Upper Q, ≥ 75th percentile for platelet count

Figure S3. Beta diversity distances from the lower 3 quartile groups using different measurement methods in the whole subjects population

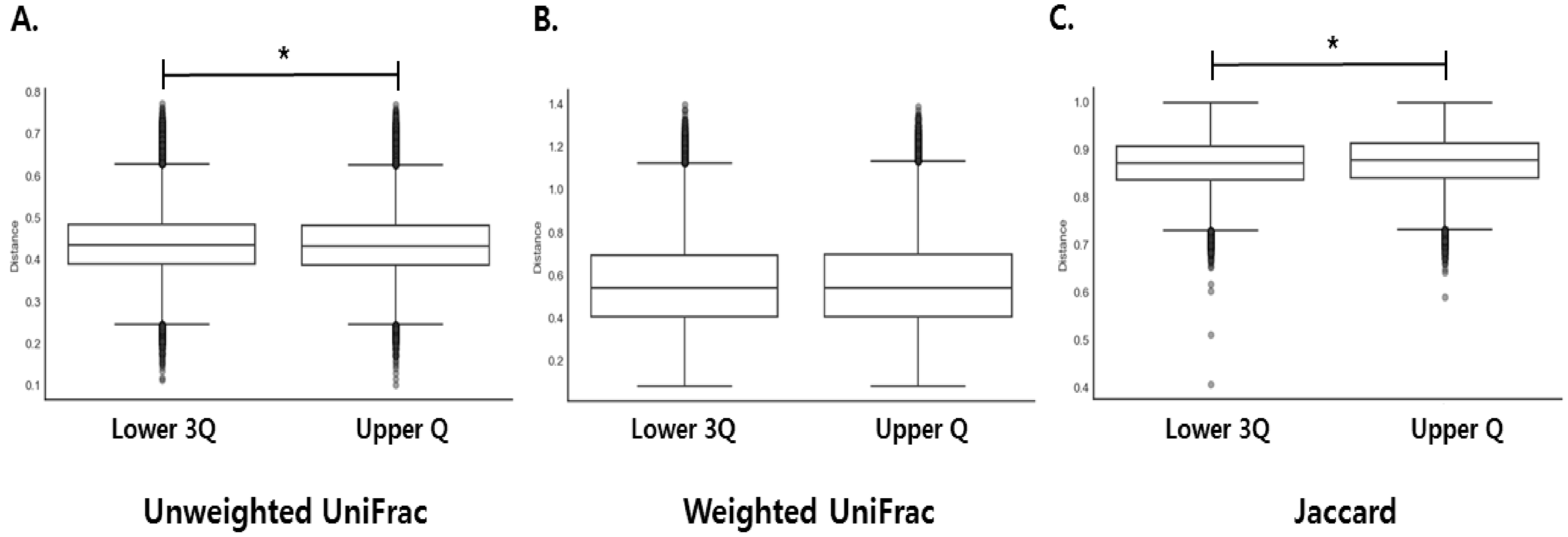

(A) Unweighted UniFrac distance (B) weighted UniFrac distance (C) Jaccard dissimilarity

Lower 3Q, < 75th percentile for platelet count; Upper Q, ≥ 75th percentile for platelet count

*$q < 0.05$,

Figure S4. Beta diversity distances from the lower three quartile groups using different measurement methods in males

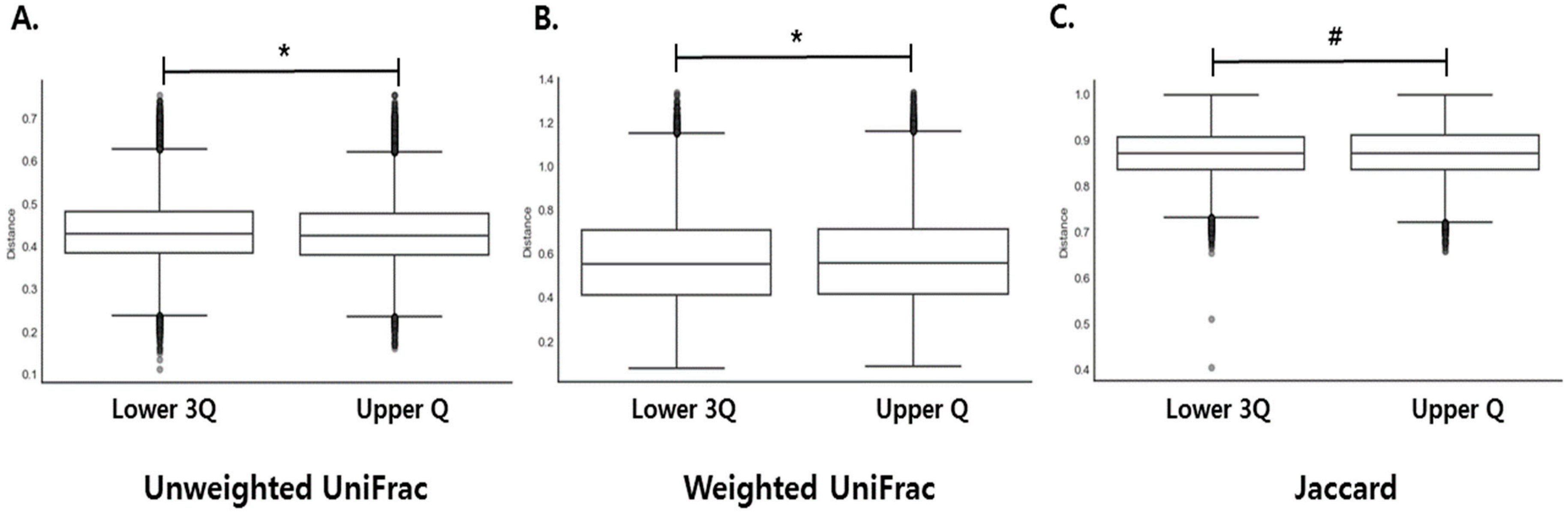

(A) Unweighted UniFrac distance (B) weighted UniFrac distance (C) Jaccard dissimilarity

Lower 3Q, < 75th percentile for platelet count; Upper Q, ≥ 75th percentile for platelet count

*q < 0.05, #q < 0.1

Figure S5. Beta diversity distances from the lower here quartile groups using different measurement methods in females

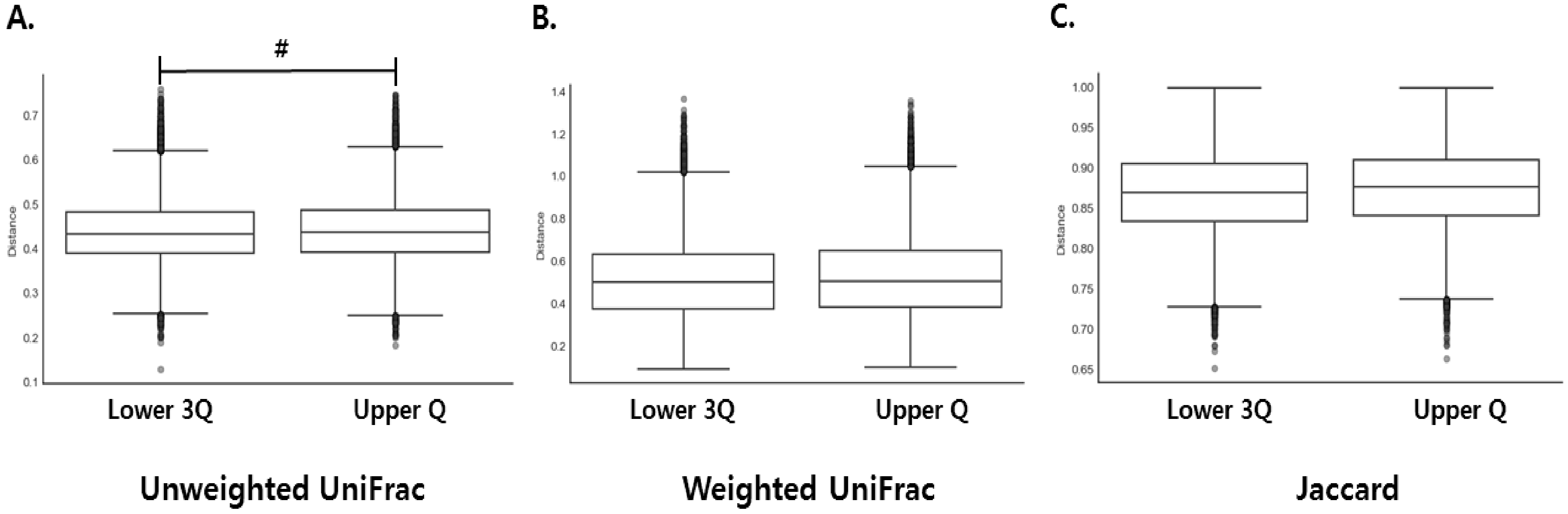

(A) Unweighted UniFrac distance (B) weighted UniFrac distance (C) Jaccard dissimilarity

Lower 3Q, < 75th percentile for platelet count; Upper Q, ≥ 75th percentile for platelet count

#q<0.1

Figure S6 Prediction of metagenome functional content correlated with platelet count using PICRUSt

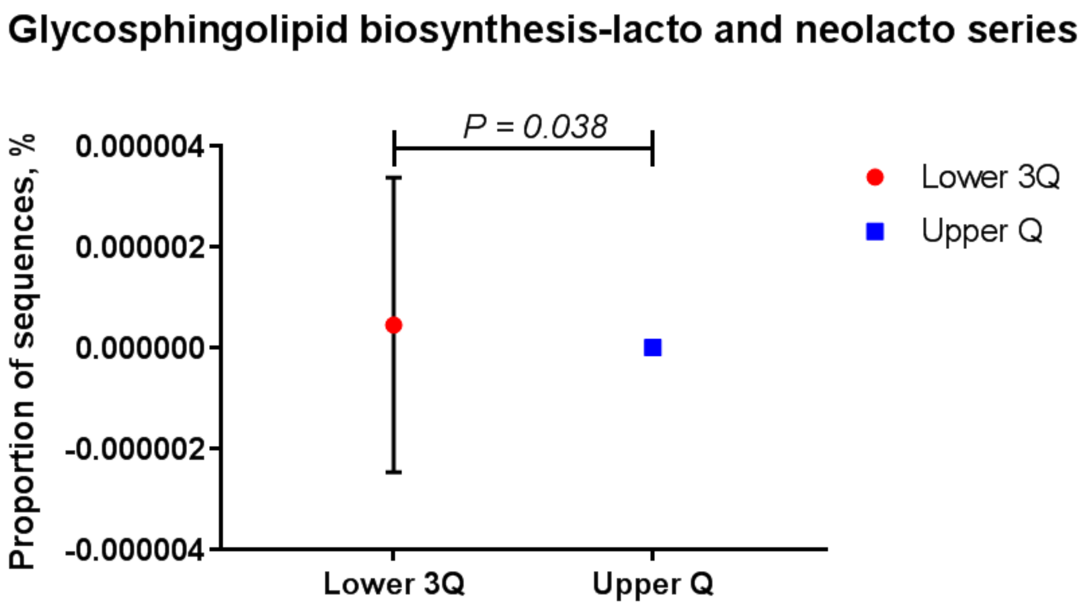

(A) A dot and error bars showing the distribution in the proportion of specific pathways assigned to samples from the upper Q and lower 3Q groups. 75th to 25th of the data). The mean value with standard errors is shown as a dot with error bars.

PICRUSt, Phylogenetic Investigation of Communities by Reconstruction of Unobserved States; Lower 3Q, < 75% of platelet count; Upper Q, ≥ 75% of platelet count
